# Emerging Evidence of the Gut Microbiome in Chemotherapy: A Clinical Review

**DOI:** 10.3389/fonc.2021.706331

**Published:** 2021-09-16

**Authors:** Byeongsang Oh, Frances Boyle, Nick Pavlakis, Stephen Clarke, Alex Guminski, Thomas Eade, Gillian Lamoury, Susan Carroll, Marita Morgia, Andrew Kneebone, George Hruby, Mark Stevens, Wen Liu, Brian Corless, Mark Molloy, Towia Libermann, David Rosenthal, Michael Back

**Affiliations:** ^1^Northern Sydney Cancer Centre, Royal North Shore Hospital, St Leonards, NSW, Australia; ^2^Cancer Care Service, Mater Hospital, North Sydney, NSW, Australia; ^3^Faculty of Medicine and Health, University of Sydney, Sydney, NSW, Australia; ^4^University of Kansas Medical Center, Kansas City, KS, United States; ^5^Bowel Cancer and Biomarker Laboratory, Faculty of Medicine and Health, University of Sydney, Sydney, NSW, Australia; ^6^Beth Israel Deaconess Medical Center (BIDMC) Genomics, Proteomics, Bioinformatics and Systems Biology Center, Beth Israel Deaconess Medical Center, Boston, MA, United States; ^7^Harvard Medical School, Boston, MA, United States

**Keywords:** gut microbiome, chemotherapy, cancer, biomarker, adverse events

## Abstract

Increasing evidence suggests that the gut microbiome is associated with both cancer chemotherapy (CTX) outcomes and adverse events (AEs). This review examines the relationship between the gut microbiome and CTX as well as the impact of CTX on the gut microbiome. A literature search was conducted in electronic databases Medline, PubMed and ScienceDirect, with searches for “cancer” and “chemotherapy” and “microbiome/microbiota”. The relevant literature was selected for use in this article. Seventeen studies were selected on participants with colorectal cancer (CRC; n=5), Acute Myeloid Leukemia (AML; n=3), Non-Hodgkin’s lymphoma (n=2), breast cancer (BCa; n=1), lung cancer (n=1), ovarian cancer (n=1), liver cancer (n=1), and various other types of cancers (n=3). Seven studies assessed the relationship between the gut microbiome and CTX with faecal samples collected prior to (n=3) and following CTX (n=4) showing that the gut microbiome is associated with both CTX efficacy and toxicity. Ten other prospective studies assessed the impact of CTX during treatment and found that CTX modulates the gut microbiome of people with cancer and that dysbiosis induced by the CTX is related to AEs. CTX adversely impacts the gut microbiome, inducing dysbiosis and is associated with CTX outcomes and AEs. Current evidence provides insights into the gut microbiome for clinicians, cancer survivors and the general public. More research is required to better understand and modify the impact of CTX on the gut microbiome.

## Introduction

Chemotherapy (CTX), systemic cancer treatment involving cytotoxic drugs, has significantly improved the overall survival of people with cancer (1). However, the main drawback of CTX are adverse events (AEs) related to treatment, which impact on both the physical and psychological well-being of patients. Up to 87% of people experienced at least one AE during and after CTX (2, 3), although recent advancements in CTX have achieved more tolerable and safer outcomes. Common AEs are nausea and vomiting, bloating, diarrhea, constipation, mucositis, CTX-induced peripheral neuropathy (CIPN), fatigue, hot flushes, anxiety and depression, insomnia and cognitive impairment. CTX also has the effect of suppressing immune responses and increasing the incidence of infection and subsequent morbidity and mortality (4).

Despite clinical practice guidelines that summarise evidence of effective strategies for preventing and managing AE’s (5–7), implementation has proven challenging. A further challenge for managing AEs, is to minimise health service costs (8–10), and also minimise the financial burden for patients and their families arising from cancer treatment (4, 11, 12). Furthermore, there are few effective biomarkers that have been developed to predict and/or proactively manage CTX-induced AEs (13, 14).

In the past decade, numerous studies have reported that the gut microbiome, defined as the collection of genomes from all micro-organisms in a given environment (15), is associated with the pathogenesis of cancer, including breast, colorectal (CRC), ovarian, and prostate (PCa) cancers (16). For example, an exploratory study in breast tissue in women with breast cancer examined the microbiota, defined as all the micro-organisms found in the environment and a term that is often used interchangeably with the microbiome (15), and found that malignancy was related to the enrichment in taxa of lower abundance, including the genera *Fusobacterium, Atopobium, Gluconacetobacter, Hydrogenophaga* and *Lactobacillus* (17). Another study compared differences in the gut microbiome of patients with CRC and healthy populations and found that the relative abundances of *Prevotella, Collinsella* and *Peptostreptococcus* were significantly higher in CRC patients, whereas the relative abundance of *Escherichia-Shigella* was significantly lower (16).

Previous reviews included preclinical and clinical trials (18) with systematic therapies (19), suggesting that the gut microbiome is not only associated with the development of cancer but also with CTX-induced toxicities. Studies in melanoma patients have identified that response to immunotherapy may be modified by the gut microbiome (20, 21). Other studies have proposed that the modulation of the gut microbiome in cancer patients before and during CTX might reduce the incidence of AEs and improve the efficacy of CTX (18, 22).

To date, few studies have examined the impact of CTX on the gut microbiome of cancer survivors in relation to CTX-induced AEs. Most of the previous reviews have included both preclinical and clinical studies and attempted to elucidate the underlying mechanisms of dysbiosis of gut microbiota in cancer pathogenesis but did not examine relationships between CTX-related AEs and the gut microbiome during and after treatment. Hence, our current review assesses the impact of CTX on the gut microbiome and CTX-related AEs in cancer patients and provides meaningful information for clinicians, patients, caregivers and the general public.

## Method

A literature search was conducted using the electronic databases Medline, PubMed and ScienceDirect, with the main search terms including “cancer” and “chemotherapy” and “microbiome/microbiota”. Inclusion criteria in the searches were: clinical trials conducted with adults (> 18 years) and published in English. References contained in the included studies were carefully reviewed for relevant papers that may have been missed by electronic searches. The search strategy was performed for studies published up to November 2020.

## Results

Seventeen studies were identified from the three electronic databases (Medline, PubMed and ScienceDirect) and included in this review (**Table 1**).

**Table 1:** The gut microbiome in chemotherapy outcomes.

1.1 Baseline Gut Microbiome in relation to Chemotherapy Outcomes						
Study Year Country	Cancer Type Sample size (n) Age	Chemotherapy (CTX)	Antibiotic Use	Faecal Sample Collection Microbiome Analysis	Outcomes measurement	Results
Zhao et al. (23) 2020 China	Advanced lung cancer (n= 64) 60 yrs (33–78)	CTX Pemetrexed + platinum ± bevacizumab (n=34) Paclitaxel/gemcitabine + platinum (n=12) Etoposide + platinum (n=18)	Excluded antibiotic users prior to CTX	1x (prior to chemotherapy) Metagenome	Association between the gut microbiome and CTX outcomes *Adverse events after chemo* Responders (R) (n=33) Non-responders (NR) (n=31)	Responders vs Non-responders NS Alpha diversity NS Beta diversity Responders ↑ *Streptococcus mutans(s)* ↑ *Enterococcus casseliflavus (s)* ↑ *Acidobacteria* *↑ Granulicella* Non-respondersi ↑ *Leuconostoclactis* *(s)* ↑ *Eubacteriumsiraeum (s)* ↑ *Streptococcus oligofermentans*, ↑ *Megasphaeramicronuciformis*, ↑ *Eubacteriumsiraeum* ↑ *Rothiadentocariosa* Adverse events after chemo ↑ *Bacteroidesnordii* ↑ *Ruminococcus sp_5_1_39BFAA* *↓ Gardnerellavaginalis*
Uzan-Yulzari. et al. (24) 2020 Israel	BCa and gynaecology cancer (n=33) Age range (18-75) BCa (n=28) Ovarian/ endometrial cancer (n=5)	Neoadjuvant and adjuvant CTX	Collected data on use of antibiotics before CTX but excluded antibiotic user during CTX	1x before chemotherapy Metagenome	Gut microbiome and weight gain No weight gain (n=9) Weight gain (n=7)	Weight gain ↑ Alpha diversity ↑ *Erysipelotrichaceae* (c) (baseline gut bacteria) Beta diversity –significant difference between weigh gain and no gain.
Lida et al. (25) 2019 Japan	HCC (n=32)	CTX IFN, 5‐FU and cisplatin	Administered antibiotics before and during chemo	1x Before chemo V3-V4 region of 16S rRNA gene sequencing	Gut microbiome on PFS and OS	↑ PFS and OS ∞ ↑ *Blautia* ↑ OS ∞*↓ Acidaminococcus* *Antibiotic* *↓ Blautia* Relative abundance of *Blautia* in faecal microbiota before CTX was positively correlated with both PFS and OS whereas abundance of *Acidaminococcus* was negatively correlated with OS. *Blautia* were less abundant in patients who had received carbapenem before CTX
**1.2 Change of Gut Microbiome in Pre-Post Chemotherapy**						
Shuwen et al. (26) 2020 China	N=25 Advanced CRC (n=11) Stage II-III Postoperative CRC (n=15)	FOLFIRI regimen (n=11) for stage IV CRC FOLFIRI regimen plus cetuximab (n=4) Adjuvant CTX XELOX regimen (n=15) for stage II-III CRC	No data	2 x (pre and post chemo) V4 region of 16S rRNA gene sequencing	Change of pre- post XELOX regimen in gut bacteria Change of pre-post FOLFIRI regimen in gut bacteria FOLFIRI + cetuximab vs FOLFIRI	Pre-post XELOX regimen *↑ Veillonella* *↓ Prevotella* *↓ Faecalibacterium* *↓ Clostridiales* Pre-post FOLFIRI regimen *↓ Faecalibacterium* *↓ Clostridiales* *↓ phascolarctobacterium* *↓ Humicola* *↓ Rhodotorula* *↑ Candida* *↑ Magnusiomyces* *↑ Tremellomycetes* *↑ Dipodascaceae* *↑ Saccharomycetales* *↑ Malassezia* *↑ Lentinula* FOLFIRI regimen plus cetuximab vs FOLFIRI regimen alone *↓ Humicola, Rhodotorula* *↓ Rhodotorula*, *↓ Magnusiomyces*
Tong et al. (27) 2020 China	Ovarian Cancer (n=18) 56 yrs	Radical surgery plus CTX (carboplatin, paclitaxel, cisplatin)	No data	8 x Preoperative, 4 weeks after surgery, before CTX, and after the first to fifth cycles of chemotherapy V4-V5 region of 16S rRNA gene sequencing	Effect of CTX on gut microbiome in ovarian cancer patients	Preoperative vs Postoperative Postoperative *↓* Bacteroidetes (p) *↓* Firmicutes (p) ↑ Proteobacteria (p) *↓ Bacteroides* (g) *↓ Faecalibacterium* (g) *↓ Bilophila* (g) *↓ Collinsella* (g) *↓ Coprococcus* (g) ↑ *Enterobacter* (g) ↑ *Klebsiella* (g) ↑ *Enterococcus* (g) Before CTX ↑ *Proteobacteria (p)* Post CTX *↑ Bacteroidetes* *↑ Firmicutes* *↑ Blautia* *↓ Proteobacteria (p)*
Galloway-Peña et al. (28) 2020 USA	AML (n =97) 58 yrs (21–85)	Induction CTX (IC)	Received prophylactic antimicrobials during IC	Oral swabs and faecal samples > 3x Biweekly from baseline until neutrophil recovery following induction CTX (IC) V4 region of 16S rRNA gene sequencing	Increased risk for infections	Effect of chemo on microbiome Baseline microbiome related to infection risk *↓* α-diversity *↓ Porphyromonadaceae* Baseline microbiome related to infection free ↑ α-diversity *↑ Porphyromonadaceae (f)* *↑ Lachnospiraceae (f)* During and Post IC *↓* α-diversity (both oral and faecal samples) *↓ Clostridiales* *↓ Blautia* ↑ *Staphylococcus* Post IC related with risk of infection Antibiotics receipt of a carbapenem>72 hours *↓* α-diversity *↓ Clostridiales* *↓ Ruminococcaceae* *↓ Porphyromonadaceae*
Kong et al. (29) 2019 China	CRC (n=43) Stage II, III and IV	AC regimen (CapeOx) Pre-surgery (n=19) Post-surgery (n=10) CTX(n=45)	No data	7x Pre-surgery Post-surgery CTX(5 x after each cycle) V4-V5 region of 16S rRNA gene sequencing	Pre vs post-surgery in gut bacteria AC regimen (CapeOx)	Pre vs post-surgery Post-surgery NS Alpha diversity *↓* ratio of Bacteroidetes to Firmicutes *↓ Bacteroidetes (p)* *↓ Bacteroides (g)* *↓ Parabacteroides* (g) *↓ Faecalibacterium* (g) *↓ Prevotella_9 (g) ↓ Bifidobacterium (g)* *↓ Fusobacterium*, *↑ Firmicutes (p)* *↑ Proteobacteria (p)* ↑ *Escherichia-Shigella*(g) ↑ *Enterobacteriaceae_unclassified*(g) ↑ *Veillonella*(g) ↑ *Morganella*(g) ↑ *Streptococcus*(g) ↑ *Proteus*(g) ↑ *Blautia* (g) *↑ Lactobacillus* (g) *↓ Enterococcus* (g) *↓ Bacillus*(g) *↓ Bilophila*(g) *↓ Barnesiella*(g) Pre vs post CTX NS Alpha diversity ↑ ratio of Bacteroidetes to Firmicutes ↑ *Bacteroidetes* *↓ Morganella* *↓ Pyramidobacter * *↓ Proteus* *↓ Escherichia-Shigella * ↑ *Bilophila* ↑ *Comamonas* ↑ *Collinsella* ↑ *Butyricimonas* ↑ *Eggerthella* ↑ *Anaerostipes *
Galloway-Peña et al. (30) 2017 USA	AML (n =55)	IC	Received prophylactic antimicrobials before and during IC	> 3x Baseline: before or within first 24h of CTX; Follow-up: every 96h until neutrophil recovery V4 region of 16S rRNA gene sequencing	Risk of infection	Risk of infection ↑ intra-patient temporal variability of α-diversity (CV of Shannon) ↑ *Staphylococcus* ↑ *Streptococcus* ↑ *Akkermansia* ↑ *Subdilogranulum* ↑ *Pseudobutyrivibrio*
Galloway-Peña et al. (31) 2016	AML (*n* = 34) 55yrs	IC	Received prophylactic antimicrobials during IC	> 3x Baseline: before or within first 24h of CTX; Follow-up: every 96h until neutrophil recovery V4 region of 16S rRNA gene sequencing	Increased risk for infections	*Baseline data associate with risk of infection* *↓* baseline α-diversity ↑ *Stenotrophomonas (genus)* Change of microbiome during and after Chemo *↓* α-diversity *↑ Lactobacillus (g)* *↓ Blautia (g)* *↓ Prevotella (g)* *↓ Leptotrichia (g)* Antibiotic Cabapenem user *↓* α-diversity
Montassier et al. (32) 2015 France	Non-Hodgkin’s lymphoma (*n* = 28) 55 yrs (45–62) Previous CTX history (n=27)	CTX Myeloablative conditioning regimen for 5 consecutive days, including high‐dose Carmustine (Bis‐chloroethylnitrosourea),Etoposide, Aracytine and Melphalan.	Most of patients (n=24, 86%) received antibiotic prophylaxis before CTX but not during CTX	2x Baseline before CTXand follow-up V5 and V6 region of 16S rRNA gene sequencing	CTX-induced changes in the gut microbiome	Pre and Post CTX ↓ α-diversity (Faith’s phylogenetic diversity, observed species) Beta- diversity (significant ↑ *Proteobacteria (p)* ↑ *Citrobacter* (g) ↑ *Klebsiella* (g) ↑ *Enterococcus* (g) ↑ *Megasphaera* (g) ↑ *Parabacreroides* (g) ↓ Firmicutes (p) ↓ Actinobacteria (p) ↓ *Ruminococcus* (g) ↓ *Oscillospira* (g) ↓ *Blautia* (g) ↓ *Lachnospira* (g) ↓ *Roseburia* (g) ↓ *Dorea* (g) ↓ *Coprococcus* (g) ↓ *Anaerostipes* (g) ↓ *Clostridium* (g) ↓ *Collinsella* (g) ↓ *Adlercreutzia* (g) ↓ *Bifidobacterium*(g)
Montassier et al. (33) 2014 France	Non-Hodgkin’s lymphoma (*n*= 8) 50 yrs	CTX 5 days of high-dose CTX: Carmustine (bis-chloroethylnitrosourea), etoposide, aracytin and melphalan	7 patients received antibiotic prophylaxis before chemo	2x Baseline: before CTX; Follow-up: 1 week after CTX V5/V6 region of 16S rRNA gene sequencing V6/V8 region of 16S rRNA genedHPLC	CTX-induced changes in the gut microbiome	↓ α-diversity ↑ *Bacteroidetes (p)* ↑ *Bacteroides* (g) ↑ *Escherichia* (g) ↓ *Firmicutes (p)* *↓ Actinobacteria (p)* ↓ *Blautia* (g) ↓ *Faecalibacterium* (g) ↓ *Roseburia* (g) ↓ *Bifidobacterium* (g)
Stringer et al. (34) 2013 Australia	Total participants (n=28) Cohort 1 Variety of cancer (n=16) 71 yrs (36–82) Healthy control (n=2) Cohort 2 (n=10) 63 yrs (40–77) Healthy control (n=5)	CTXin Cohort 1: Capecitabine, cisplatin/5-FU, FOLFOX, 5-FU/folinic acid, COFF and paclitaxel, carboplatin and gemcitabine CTXin Cohort 2: (FOLFOX4, FOLFOX6, FOLFIRI, capecitabine)	Most of patients received antibiotics prior to collection of a faecal sample.	4x (Before chemotherapy, After first chemo Day 2, Day 5, and Day 10) qRT-PCR	CTX-induced diarrhoea (CID) Cancer pts vs healthy control Change of gut bacteria in CID	Cohort 1: Cancer pts vs Healthy control ↓ *Lactobacillus spp*. *↓ Bifidobacteriumspp* *↓ Bacteroides spp*. *↓ Enterococcus spp*. ↑ *Escherichia coli* ↑ *Staphylococcus* spp. Cohort 2: Change of gut bacteria in CID ↑ *E.coli* *Lactobacillus spp*. (up to day 5↑, then decrease Day 10 ↓
Zwielehner et al. (35) 2011 Austria	Total participants (n=34) Various Cancers (n=17) 59 yrs Healthy control (n=17) 65 yrs	CTX (n=11) CTX+ Antibiotics (n=6)	CTX plus antibiotics	4x (Before CTX, Day 1–4 after CTX and Day 5–9 after CTX) qPCR/PCR-DGGE 16S rRNA genes	Patients with CTX +antibiotics vs healthy control Differential changes in the gut microbiome: CTX vs CTX + antibiotics	Patients with CTX + antibiotics vs healthy control ↓ Absolute bacterial numbers ↓ *Clostridium* clusters *IV* and *XIVa* ↓ *Bacteroides* ↓ *Bifidobacteria* ↓ *Clostridium* cluster *IV* CTX + antibiotics vs CTX NS *Clostridium* cluster *XIVa* ↑ *Clostridium difficile* During CTX ↑ *Bacteroides* spp. ↓ *Bacteroides* ↓ *Bifidobacteria* ↓ *Clostridium* cluster *IV* and *XIVa*
**1.3 Change of Gut Microbiome in Post Chemotherapy**						
Okubo et al. (36) 2020 Japan	Stage I-III BCa (n= 126) Mean age 58 yrs	CTX (n=57) No CTX (n=69)	No data	1x Follow up visit V3-V4 region of 16S rRNA gene sequencing	Fear of cancer recurrence (FCR) CTX vs no CTX	Higher FCR *↓ alpha diversity* *↓ Firmicutes (p)* *↑ Bacteroidetes (p)* *↑ Bacteroides (g)* *↓ Lachnospiraceae (g)* *↓ Ruminococcus (g)*
Guan et al. (37) 2020 China	HER2-negative metastatic breast cancer (n=31)	Lower dose metronomic capecitabine (n=15) Conventional dose (n=16)	No data	1 x Follow-up visit after chemotherapy V4 region of 16S rRNA gene sequencing	Metronomiccapecitabine vs Conventionaldose	NS Alphadiversity Metronomiccapecitabine *↓ Cyanobacteria (p)* *↓ Blautia (g)* *↓ o_Streptophyta (g)* ↑ *Megamonas (g)* ↑ *f_Mogibacteriaceae (g)*
Fei et al. (38) 2019 China	Stage III CRC (n=17)	CTX CapeOX regimen (capecitabine twice daily combined with oxaliplatin every 3 weeks) CTX induced diarrhea (CID) (n=4) No CID (n=13)	Excluded patients with CID grade 3 and grade 4 during CTX	1x (2 weeks after 8 cycles of CTX) V3-V4 region of 16S rRNA gene sequencing	Gut bacteria related with CID	CID *↓* α-diversity ↑ *Proteobacteria* ↑ *Enterobacteriales* ↑ *Gammaproteobacteria* ↑ *Enterobacteriaceae* ↑ *Klebsiella* *Klebsiellapneumoniae* was the most predominant species (31.22%) among the gut microbiome NO CID ↑ *Clostridiales*, ↑ *Clostridia* ↑ *Ruminococcaceae* ↑ *Bacteroidetes* ↑ *Bacteroidia* ↑ *Bacteroidales* ↑ *Bacteroides* ↑ *Bacteroidaceae* .
Deng et al. (39) 2018 China	Total (n=69) C: Healthy individuals (n=33), I: before CTX CRC (n=17), D: after CRC + CTX(n=14), O: CRC + surgery (n=5)	CTX: 5 FU+ oxaliplatin (6-8 cycles)	No data	1x before CTX V4-V5 region of 16S rRNA gene sequencing	CRC plus CTX vs CRC	After CTX CRC vs CRC ↑ *Fusobacteria (p)* ↑ *Veillonella dispar (g)* ↑ *Prevotellacopri (g)* ↑ *Bacteroidesplebeius(g)* ↑ *Fusobacterium (g)* *↓ Bacteroides (g)* ↑ *Bacteroidetes (p)* ↑ *Firmicutes* *(p)*

↑, Increase; ↓, Decrease; NS, Not significant; ∞, Association; AML, Acute myeloid leukemia; CV, coefficient of variation; BCa, Breast Cancer; CRC, colorectal cancer; RCC, renal cell carcinoma; NSCLC, non-small cell lung cancer; HSCT, hematopoietic stem cell transplantation; PFS, progression-free survival; OS, overall survival; AEs, Adverse Events; CID, Chemotherapy induced diarrhea; qPCR, quantitative polymerase chain reaction; qRT/PCR, quantitative real time polymerase chain reaction; DGGE, Denaturing gradient gel electrophoresis; dHPLC, Denaturing high-performance liquid chromatography; PCR-DGGE, polymerase chain reaction denaturing gradient gel electrophoresis; 5-FU, 5-flourouracil; FOLFIRI regimen [irinotecan, leucovorin and 5-fluorouracil, Capecitabine plus oxaliplatin (CapeOx), combined 5-fluorouracil (FU)-leucovorin therapy plus either irinotecan (the FOLFIRI regimen) or oxaliplatin (the FOLFOX regimen), XELOX regimen [capecitabine and oxaliplatin].

Definition of terms used in microbiota research.

α-diversity: Number and evenness of distribution of taxa within a given sample.

β-diversity: The difference in diversity of taxa from one sample to another, i.e., the number of taxa that are not the same (or not similarly distributed) in two different samples.

16S rRNA gene: Marker gene for bacterial identification, containing evolutionary conserved universal as well as variable regions.

Operational taxonomic unit (OTU): Cluster of nearly-identical sequences (e.g., 97% similarity), often used in microbiota research instead of ‘species’.

16S rRNA gene sequencing: Sequencing of the 16S rRNA marker gene.

Metagenomic sequencing: Sequencing of the entire metagenome (all the genetic material in a sample), also allowing analysis of the functional capacity of the microbiome.

### Characteristics of Clinical Studies

The seventeen selected studies included a total of 742 patients with a range of 8 – 126 participants in each of the studies. Cancers studied included CRC (n=5) (26, 29, 38, 39), AML (n=3) (28, 30, 31), BCa (n=2) (24, 36), Non-Hodgkin’s lymphoma (n=2) (32, 33), lung cancer (n=1) (23), ovarian cancer (n=1) (27), liver cancer (n=1) (25), and various other types of cancers (n=3) (24, 34, 35). Six studies were conducted in China (23, 26, 27, 29, 38, 39), three in the USA (28, 30, 31), two in France (32, 33), two in Japan (25, 36), and one each in Austria (35), Australia (34) and Israel (24). Total CTX dosage, type of CTX drugs and periods of CTX interventions varied across studies. Study designs and the primary outcomes of individual studies included in this review were diverse.

Three studies assessed the relationship between the gut microbiome and CTX outcomes [response to CTX and AEs (23), weight gain (24), PFS and OS (25)] with faecal samples collected prior to CTX.

Eleven studies collected faecal samples multiple times (before, during and/or after CTX) and assessed the impact of CTX on the gut microbiome and the relationship between the gut microbiome and CTX-related AEs, including risk of infection, diarrhea and the effects of antibiotics (27, 28, 30–35).

In studies conducted with cancer survivors who had undergone chemoradiotherapy (CRT), two studies assessed the relationship between the gut microbiome and CTX-related AEs [fear of recurrence (36), diarrhea (38)], whereas two studies assessed the impact of CTX on the gut microbiome [low vs high dosage CTX (37), surgery vs surgery plus CTX (39)] with faecal samples collected after CTX.

Fifteen studies analysed the gut microbiome profile with the 16S ribosomal RNA (16S-rRNA) gene sequencing method, two with metagenomic sequencing (23, 24), one study with quantitative real time polymerase chain reaction (qRT/PCR) (34) and another study with qPCR and Denaturing gradient gel electrophoresis (DGGE) (35). Interestingly, analysis of gene sequencing regions of 16S rRNA varied across studies: V3-V4 (n=3) (25, 36, 38), V4 (n=4) (26, 28, 30, 31), V4-V5 (n=5) (27–29, 32, 39), V5-V6 (n=1) (33), qRT/PRC (n=1) (34), and qPCR/PCR-DGGE (n=1) (35).

#### The Gut Microbiome Prior to CTX Is Related to CTX Outcomes Including Efficacy and Toxicity

Three studies assessed the relationship between baseline composition and diversity of the gut microbiome and CTX outcomes (23–25). Two studies reported that the composition and diversity of the gut microbiome was related to the efficacy of CTX (23, 25) and one study found that the composition and diversity of the gut microbiome was related to weight gain (24).

A recent study examined the relationship between the baseline gut microbiome in lung cancer patients (n=63) and CTX outcomes and found that the baseline gut microbiome was associated with both response to CTX and AEs (23). The relative abundance of *Streptococcus mutans* and *Enterococcus casseliflavus* were higher in responders (n=33) (p < 0.05), whereas 11 gut bacteria including *Leuconostoc lactis* and *Eubacterium siraeum* were enriched in non-responders (n=31) (p < 0.05). In addition, the relative abundance of *Bacteroides nordii*, *Ruminococcus sp_5_1_39BFAA* and *Gardnerella vaginalis* were associated with severe AEs of CTX.

A recent study of patients with BCa and gynaecological cancers (n=35) assessed the relationship between the gut microbiome and weight gain in those treated with adjuvant CTX (24). The study found that higher alpha diversity and enriched composition of the microbiome in pretreatment faecal samples was associated with weight gain following CTX (24).

Furthermore, faecal microbiota transplantation (FMT) from pre-treatment samples of those patients’ who gained weight post-treatment induces, glucose intolerance, adverse lipid changes and inflammatory changes in germ-free Swiss Webster mice (24). These results suggest that the gut microbiome is mediating metabolic changes in women who undertake CTX, as an adjuvant treatment, however further examination in a larger patient cohort is warranted.

An innovative study examined the effect of antibiotic use in patients with hepatocellular carcinoma (n=32) during CTX and found that the use of antibiotics (carbapenem) before and during CTX was associated with poor PFS and OS (carbapenem + vs. −; median PFS, 78 days vs. 154 days, p = 0.0053; median OS, 177 days vs. 475 days, p = 0.0003) (25). Notably, in this study it was reported that the relative abundance of *Blautia* in faecal samples before CTX was positively correlated with both PFS and OS, whereas abundance of *Acidaminococcus* was negatively correlated with OS. It also found that *Blautia* were less abundant in patients who had received carbapenem before CTX.

Another study compared the composition of gut bacteria in four groups, viz. healthy controls (n=33) vs patients diagnosed with CRC (n=17) vs patients with CRC plus surgery (n=5) vs patients with CRC plus CTX (n=14) (39). This study reported that, at the genus level, *Veillonella and* species *Veillonella dispar* were observed in CRC patients treated with CTX, but not in the other three groups. In addition, although not detected at the genus level, two other species, *Prevotella copri* and *Bacteroides plebeius*, were enriched in CRC patients treated with CTX (39), whereas alpha diversity of the gut microbiome was lower in CRC patients who received surgery, compared to the other three groups.

#### Impact of CTX on the Gut Microbiome: Chemotherapy Impact on the Diversity and Composition of the Gut Microbiome

##### The Gut Microbiome and CTX Outcomes

Ten studies examined the effect of CTX on the gut microbiome in patients receiving CTX; three with AML, two each with CRC, Non-Hodgkin’s lymphoma, a mixed group of cancers, and one with ovarian cancer patients.

##### Colorectal Cancer (CRC)

Two studies conducted in CRC patients reported changes in the gut microbiome pre and post CTX (26, 29). One study examined the effects of three palliative CTX regimens (FOLFIRI (n=11) vs FOLFIRI regimen plus cetuximab (n=4) vs XELOX regimen (n=15)) on the gut microbiome and found that impacts on the gut microbiome varied according to the CTX regime administered (26). Namely, this study reported that the relative proportions of *Faecalibacterium, Clostridiales, phascolarctobacterium, Humicola and Rhodotorula* were decreased, and the abundances of *Candida, Magnusiomyces, Tremellomycetes, Dipodascaceae, Saccharomycetales, Malassezia and Lentinula* were increased in advanced CRC patients treated with the FOLFIRI regimen. In comparison with those treated with the FOLFIRI regimen alone the proportion of *Humicola, Rhodotorula, and Magnusiomyces* were decreased in advanced CRC patients treated with the FOLFIRI regimen combined with cetuximab, whilst those of *Candida, Tremellomycetes, Dipodascaceae, Saccharomycetales, Malassezia and Lentinula* were increased. The abundances of *Veillonella, Humicola*, *Tremellomycetes* and *Malassezia* were increased in postoperative CRC patients treated with the XELOX regimen. Another study conducted with CRC patients in stage II-IV (n=43) collected faecal sample five times after each treatment cycle and reported an increased ratio of *Bacteroidetes to Firmicutes*, *Bacteroidetes, Bilophila Comamonas, Collinsella, Butyricimonas, Eggerthella and Anaerostipes, and decreased Morganella*, *Pyramidobacter, Proteus*, and *Escherichia-Shigella* after CTX (29).

##### Acute Myeloid Leukemia

Three studies assessed the predictive value of the gut microbiome and its relationship to infection risk in patients with AML (28, 30, 31). Although these studies were conducted by the same research team, it was reported that the associations between the relative abundance of the gut microbiome and risk of infection varied across studies, while associations between risk of infection and alpha diversity of the gut microbiome was consistent in two studies (28, 31). One study (n=34) reported that lower alpha diversity and enriched *Stenotrophomonas* prior to CTX was associated with risk of infection in patients with AML (31). They also reported decreased alpha-diversity and decreased *Blautia*, *Prevotella, Leptotrichia* of the microbiome but increased *Lactobacillus* at the genus level during and after CTX. A subsequent study conducted with AML patients (n= 55) reported that increased intra-patient temporal variability of alpha-diversity and enriched *Staphylococcus, Streptococcus*, *Akkermansia*, *Subdilogranulum*, and *Pseudobutyrivibrio* were associated with risk of infection (30). The third study conducted with a large sample size (n=97) reported that, at baseline, higher alpha-diversity (hazard ratio [HR], 0.36; 95% confidence interval [CI],.18–.74) and relative abundance of *Porphyromonadaceae* (HR, 0.36; 95% CI,.18–.73) were associated with an increased probability of remaining infection-free during neutropenia (28). This study reported that the use of antibiotics (carbapenem >72 hours) increased the risk of infection in addition to lowering alpha-diversity and a relative low abundance of *Clostridiales, Ruminococcaceae* and *Porphyromonadaceae*.

##### Non-Hodgkin’s Lymphoma

Two studies examined the impact of CTX on the gut microbiome in patients undergoing one course of bone marrow transplantation (BMT) conditioning CTX and found that CTX changed the diversity and composition of the gut microbiome (32, 33).

An earlier study conducted with a small sample size (n=8) reported a significant reduction in alpha diversity and alterations in the composition of the gut microbiome associated with GI toxicities, viz. decreased *Faecalibacterium, Bifidobacterium* and increased *Bacteroides, Proteobacteria* and *Escherichia (*33*)*. A subsequent study by the same researchers compared the diversity and composition of the gut microbiome with faecal samples from patients (n=28) before and after CTX (32). They reported, at genus level, significant decreases in the abundance of *Ruminococcus, Oscillospira, Blautia, Lachnospira, Roseburia*, *Dorea, Coprococcus, Anaerostipes, Clostridium, Collin- sella, Adlercreutzia and Bifidobacterium*.

##### Ovarian Cancer

One study assessed changes in the gut microbiome of ovarian cancer patients pre and post-surgery compared with surgery plus CTX (27). This study found significant decreases in the abundance of *Bacteroidetes* and *Firmicutes* and increases in the abundance of *Proteobacteria* after surgery. Interestingly, a comparison of pre and post CTX following surgery found the abundance of *Bacteroidetes* and *Firmicutes* increased, and the abundance of *Proteobacteria* decreased after CTX.

Furthermore, some forms of anaerobic bacteria, such as *Bacteroides, Collinsella* and *Blautia* significantly increased after CTX.

##### Mixed Group of Cancers

Two earlier studies explored the effects of CTX on gut bacteria in people diagnosed with various cancers (34, 35). One study examined the effect of gut bacteria related to CTX-induced diarrhea (CID) (34). They compared the gut bacteria of cancer patients with CID and a healthy control group and found decreased *Lactobacillus* spp.*, Bifidobacterium* spp.*, Bacteroides* spp. and *Enterococcus* spp. and increased *Escherichia coli and Staphylococcus* spp. in patients with CID (34).

Another similar study compared differences between patients after CTX and a healthy group, and found decreased diversity and *Clostridium* clusters *IV* and *XIVa*, *Bacteroides*, *Bifidobacteria*, *Clostridium* cluster *IV* in patients after CTX (35). In addition, this study investigated the differences in gut bacteria between patients who received CTX with antibiotics and those without and found an abundance of *Clostridium difficile* in patients treated with antibiotics. In these two studies, faecal samples were analysed using conventional culture techniques and qRT-PCR, while other studies included in this review were analysed using next generation sequencing (NGS), and advanced technology including 16S rRNA sequencing and metagenomics.

#### The Gut Microbiome in Cancer Survivors With a History of CTX

Four studies examined the relationship between the gut microbiome and cancer survivorship in patients who had received CTX (36–39). Faecal samples were collected at the follow-up visit after CTX and examined the relationship between the gut microbiome and post CTX-related AEs. Two studies examined the effects of CTX on the gut microbiome (low dose vs traditional dose of CTX and PFS (37), surgery vs surgery plus CTX) (39), and two other studies assessed the relationship between CID (38) and fear of recurrence (36) after CTX.

##### Chemotherapy-Induced Diarrhea (CID): The Microbiome Is Associated With CID

An earlier study explored the association between the gut microbiome and CID (completed 8 cycles of the CapeOX regimen: capecitabine (1000 mg/m^2^ twice daily) combined with oxaliplatin (130 mg/m^2^ every 3 weeks) in patients diagnosed with stage III CRC. This study found that the gut microbial community richness and diversity were lower after CTX in CID (p < 0.05) compared to a control group (38). It also reported that, at the species level, there were significant differences in 75 micro-organisms between the CRC with and without CID and identified that *Klebsiella pneumoniae* was the most predominant species among the gut microbiome in patients with CID.

##### Lower Dose Versus Conventional Dose: The Microbiome Is Associated With PFS

A recent study examined the effect of CTX (low doses of metronomic capecitabine (n=15) vs conventional doses (n=16)) on the gut microbiome in women diagnosed with HER2-negative metastatic breast cancer and found that beta diversity (unweighted-unifrac index) was significantly lower in the metronomic group compared to the group who received conventional doses (p=0.025) (37). Furthermore, they found that the median PFS was significantly shorter in patients with the gut bacteria composition of *Slackia* (9.2 vs. 32.7 months, *P* = 0.004), while the patients with *Blautia obeum* had a significantly prolonged PFS compared to those without (32.7 vs. 12.9 months, *P* = 0.013). At the phylum level, the main composition of *Cyanobacteria* was significantly lower in the metronomic group, while the phyla of *Bacteroidetes, Firmicutes, Proteobacteria, and Actinobacteria* were similar between the groups. In addition, at the genus level, *Megamonas* and *f_Mogibacteriaceae* were significantly enriched and *Blautia* and *o_Streptophyta* were depleted in the metronomic group.

##### Fear of Cancer Recurrence: The Microbiome Is Associated With FCR

A recent study examined the relationship between the gut microbiota and FCR in women diagnosed with invasive breast cancer with a history of CTX (n=57) and those with no history of CTX (n=60) (36). Interestingly, this study found that in women with a history of CTX and FCR, lower alpha diversity, lower relative abundance of the *Firmicutes* (p=0.03) and higher relative abundance of *Bacteroidetes* (p=0.04) at the phylum level, and higher relative abundance of *Bacteroides* (p<0.01) and lower relative abundance of *Lachinospitaceae* (p=0.03) and *Ruminococcus* (p=0.02) at the genus level, were associated with FCR while there was no significant association in women diagnosed with invasive breast cancer with no history of CTX.

##### CRC Patients With Surgery Plus CTX Versus Surgery: CTX May Impact on the Gut Microbiome

One study compared the composition of gut bacteria in four groups, viz., healthy controls (n=33) vs patients diagnosed with CRC (n=17) vs patients with CRC plus surgery (n=5) vs patients with CRC plus CTX (n=14) (39). This study reported that, at the genus level, *Veillonella and* species *Veillonella dispar* were only found in CRC patients treated with CTX but not in the other three groups. In addition, although not detected at the genus level, two other species, *Prevotella copri* and *Bacteroides plebeius*, were enriched in CRC patients treated with CTX, whereas alpha diversity of the gut microbiome was lower in CRC patients who received surgery compared to the other three groups.

#### Use of Antibiotics

Often prospective studies, seven studies collected data about use of antibiotics in participants. Three studies conducted with AML (28, 30, 31) and two studies with Non-Hodgkin’s lymphoma (32, 33) received antibiotics as prophylaxis prior and during CTX, one study each received antibiotics before chemotherapy and during CTX (34, 35). One study examined the difference between CTX with antibiotics versus CTX with no antibiotics and showed decreased diversity of *Clostridium* cluster IV and XIVa in response to CTX with cluster IV diversity being particularly affected by antibiotics (35).

Of the seven studies that examined the relationship, two studies excluded antibiotic users during recruitment (23, 38), one study included participants using antibiotics before but not during CTX (24), whereas, another study included participants using antibiotics before and during CTX (25).

## Discussion

The significance of the current review is that we not only assessed literature examining the relationship between the gut microbiome and CTX outcomes and AEs, but also the impact of CTX on the gut microbiome during treatment. Previous reviews examined the association between the gut microbiome and cancer survivors using limited clinical trials and attempted to elucidate the mechanisms of pathogenesis of cancer with preclinical studies (40, 41). Of seventeen studies reviewed, we found that three studies assessed the relationship between the gut microbiome and CTX outcomes (respondent vs non respondent, PFS, OS and weight gain) with faecal samples collected prior to CTX (23–25), whereas four other studies assessed the relationship between the gut microbiome and CTX outcomes (diarrhea, FCR, low dosage vs conventional dosage, surgery vs surgery plus CTX) with faecal samples collected either after CTX or in patients with a history of CTX (36) (37–39). In addition to evaluating these relationships, in ten other prospective studies, faecal samples were collected multiple times during CTX, and examined the impact of CTX on the gut microbiome which provided valuable insight into the importance of the gut microbiome in cancer survivors (26–35). The findings of the current review regarding the association between the gut microbiome and CTX-related AEs and treatment outcomes are consistent with previous reviews (19, 40, 41). Several studies reported consistent relationships between dysbiosis of the microbiome and CTX-related AEs and suggested that the gut microbiome has the potential to be applied as a biomarker to predict CTX outcomes and related AEs (40, 41). However, associations do not represent causation and further well-designed studies are required, such as a recent high quality clinical trial that is being undertaken in Canada exploring the effect of CTX on the gut microbiome (42). In recognition of weaknesses in the evidence from cohort studies, we examined causal links between the gut microbiome and CTX in ten prospective studies. A review of these ten studies assessed the impact of CTX on the gut microbiome during CTX and indicated that CTX modulated the gut microbiome, and that this modulatory effect is associated with an increased risk of infection and impacts on the efficacy of CTX. The ten prospective studies demonstrated the vital role of the gut microbiome in CTX and suggests that the modulation of the gut microbiome during CTX may reduce the risk of AEs and increase the efficacy of CTX. This hypothesis was partially supported by previous studies conducted with lifestyle interventions including prebiotics and exercise although there were some discrepancies among the studies (43–45).

Furthermore, several recent studies evaluated the effects of antibiotic exposure on cancer risk and reported that antibiotics tend to increase cancer risk (46–48) and reduce the efficacy of various forms of cancer therapy, including CTX, radiotherapy and immunotherapy (46, 49). Along the lines of these studies, we also assessed the use of antibiotics in this review. Of seventeen studies, nine studies (prospective studies (n=7) and cohort studies (n=2)) reported the use of antibiotics in their study design. In the prospective studies, three studies were conducted with AML (28, 30, 31) and two studies with Non-Hodgkin’s lymphoma (32, 33) where patients received antibiotics as prophylaxis prior to, and during CTX, and in one study each patient received antibiotics before CTX and during CTX (34, 35).

This study finding the association between the use of antibiotic and poor efficacy is comparable to an earlier study performed with patients with advanced non-small cell lung cancer (NSCLC) treated with nivolumab and with antibiotic (50). Previously, only one study had examined the difference in outcomes between CTX with antibiotics versus CTX with no antibiotics and reported a decreased diversity of *Clostridium* cluster IV and XIVa in response to CTX, with cluster IV diversity being particularly affected by antibiotics (35). However, a major limitation of this finding was that faecal samples were analysed using conventional culture techniques and qRT-PCR, whereas in most of the recent studies included in this review, samples were analysed using next generation sequencing (NGS) advanced technology including 16S rRNA sequencing and metagenomics.

Consequently, in future studies, a comparison of the effects of CTX on the gut microbiome of patients, with and without antibiotics, utilizing modern NGS technology will be worthwhile.

This review has several limitations. Firstly, while the main strength of the review is its assessment of causal effects of CTX on the gut microbiome, in addition to the relationships between the gut microbiome and CTX outcome and AEs, we found that a number of studies (n=10) were conducted with heterogeneous cancer populations; AML (n=3), CRC (n=2), Non-Hodgkin’s lymphoma (n=2), ovarian cancer (n=1) and mixed cancer groups (n=2). Hence, in order to identify specific gut bacteria related to certain cancer types, more studies are required with matching diagnoses for types of cancers. Also, most of the studies reviewed (n=8) were conducted with very small sample sizes ranging from 8 to 43 participants. Although two studies were conducted with moderate sample sizes of 55 and 97 participants, neither study described any power calculation in their methodologies leading to concerns about the validity of the results. Moreover, interpretations of causal effects in the outcomes of several studies are complex because of a range of potentially confounding variables. For example, administering various CTX drugs among studies instead of standard CXT drug interventions, the use of groups of multiple cancer survivors, a lack of standardization in the microbiome analysis of faecal samples, varied outcome measures and varied study design complicated the conclusions drawn. Although having studies conducted across several countries [China (n=6) (23, 26, 27, 29, 38, 39), USA (n=3) (28, 30, 31), France (n=2) (32, 33), Japan (n=2) (25, 36), Austria (n=1) (35), Australia (n=1) (34), and Israel (n=1) (24)] increases the generalizability of results, it also makes for a more complex interpretation of the data as several studies have demonstrated that the gut microbiome is associated with ethnicity, diet, physical activities and environment (51–53). Hence, future international multicentre trials will need to be conducted to provide comprehensive and reliable data that controls for confounding patient variables including age, ethnicity, gender, co-morbidities, drug use, geography, and lifestyle factors, including diet and physical activities.

Considering these limitations, future studies with larger sample sizes and robust study designs are required to provide evidence that can readily translate into the clinical setting. To our knowledge, the current comprehensive literature review is the first to examine causal relationships of the gut microbiome on CTX outcomes and AEs with prospective clinical trials, and to assess the relationship between the gut microbiome and CTX outcomes and CTX-related AEs with observational studies. In conclusion, although there is a lack of high quality clinical trials on this topic, our results from a comprehensive review provides further insight into the complex relationships between the gut microbiome and CTX and shows the potential for future research to improve patient care. The current evidence suggests the potential implications for the gut microbiome to predict CTX outcome and prevent AEs in patients undergoing CTX, however, several challenges remain and need to be resolved before recommending microbiome therapy in oncology.

## Author Contributions

All authors cooperated on developing the concept design and preparing the manuscript. Drafting of the manuscript: BO, BC, FB, TL, GH and NP. All authors contributed to the article and approved the submitted version.

## Funding

Publication fee is supported by the Department of Radiation Oncology, Royal North Shore Hospital.

## Conflict of Interest

The authors declare that the research was conducted in the absence of any commercial or financial relationships that could be construed as a potential conflict of interest.

## Publisher’s Note

All claims expressed in this article are solely those of the authors and do not necessarily represent those of their affiliated organizations, or those of the publisher, the editors and the reviewers. Any product that may be evaluated in this article, or claim that may be made by its manufacturer, is not guaranteed or endorsed by the publisher.
